# Private Hospitals

**Published:** 1921-02-05

**Authors:** 


### PRIVATE HOSPITALS.

If the seventeenth century is the era of the public hospital, this century will be chronicled by the historian as marking the development and growth of the private hospital. A general realisation of the fact that the homes of the poor were unfit habitations for the sick marked a distinct advance in scientific enlightenment. After three centuries it is beginning to be felt that a similar criticism applies to the majority of dwelling-houses, and hence we witness springing up, mushroomlike, in every city and town in the Kingdom, under the ægis of private enterprise, accommodation for sick people who can afford to pay for medical attendance and nursing. These are usually described as nursing homes. I prefer to call them private hospitals.

### A Brass Plate and a Brass Face.

What is a private hospital? In investigating a subject of this description it is desirable, if possible, to arrive at a definition. But whereas it might be simple to indicate the ideal, it is no easy task to include the real within the ambit of a common description. They are in such infinite variety. But there is one which will include thousands. Any kind of house, sanitary or otherwise; any kind of matron, skilled or unskilled; assistants ditto, ditto, with bed accommodation for the sick prey. It is, indeed, remarkable in how many respects nursing corresponds with teaching. Heaven knows how many "Dotheboys Halls" there are which bear on their door-plate the legend, "Seminary for the sons of gentlemen," or words to this effect. Anybody may open a school, and, likewise, anybody may, without breaking the law, become managing director or nurse-in-chief of a private hospital. A common lodging-house may be transformed into a private hospital in the twinkling of an eye with the aid of a brass plate and a brass face. Then the sick and afflicted may be brought in like pigeons to be plucked.

### Laissez-faire.

And yet we live in the twentieth century, in a country possessing a Ministry of Health and a Nurses' Registration Act. Did I not tell thee, friend Nurse, full many a time and oft, that this Registration Act concerning which so many trumpets sounded would make a sorry guardian of the interests of the profession? A cook and her scullery-maid, if it so pleases them, may open a private hospital, and nurse the sick for gain. Incidentally, the victims may lose their lives, but this is a detail too insignificant for the law to take notice of. The doctrine of *laissez-faire* is that the best way to cure evils is, first of all, to let them do their worst. The wildest storms abate, and the biggest fires burn themselves out. And so, a generation hence or thereabouts, it may behove the nation's health authorities to recognise the fact that the private hospital is a public scandal.

It does not by any means follow that the proprietresses of second- or tenth-rate institutions of this kind are people of malevolent design. Not at all. The temptations are strong and the difficulties inconsiderable. In the first place there is a great and a growing demand. The congestion and pressure of modern life tend to make home nursing more and more difficult. In many houses all the women are wage-earners, and even where this is not so there are multitudinous duties that cannot be allowed to suffer neglect. Thus it happens that enterprising women discover a lucrative way of living, without serious responsibility, for so long as there is a doctor in attendance awkward questions are not likely to be asked. There may be a broken leg in this room, a scarlet-fever patient next door, and a maternity case upstairs. Such a mix-up would be indiscreet, but not illegal. Whether the sanitary arrangements are sound or deadly nobody knows. In a hotel or boarding-house the guests are about, and can observe for themselves; but in an institution for the sick the patients are helpless. If they complain, their irritability is regarded as the natural accompaniment of their illness, and so they have to make the best of whatever the gods send them.

### The Commercial Motive.

If it so happens that the humanitarian spirit is to be found in a private hospital, it is there, so to speak, by chance. Not so in a public hospital, where even in the workhouse the inspiring impulse, however distant, is philanthropy. The Poor Laws are based on the principle that poverty-stricken members of the community are entitled to relief. Private hospitals are established for profit, nothing else, and the consequent tendency is to impose expense on the patient and to sweat the nurse. There is nothing derogatory in establishing an institution of this or any other kind for profit. But there is this distinction to be borne in mind, that the people who go to these hospitals for treatment are, for all practical purposes, helpless, and it therefore follows that the public are responsible for their proper administration. The supervision of the private hospital is an obvious part of the duty of the Minister of Health.

There are, it is needless to observe, numbers of private hospitals which are everything that can be desired. In making these observations I have refrained from drawing any distinction between those that are excellent and the others, because it is not possible to catalogue them separately. For this the proprietors have no remedy, and it is clearly in their immediate interests to take steps towards reform. Private hospitals should be licensed by the State and subject to inspection. To revert again to teaching, His Majesty's inspectors under the Board of Education have to see that the school buildings are suitably laid out and equipped, and that the teaching staff is properly qualified and efficient. It ought to be made an offence against the law to receive patients for medical treatment and nursing in any house not previously certified as fit by the Minister of Health. Until this is accomplished the public will be at the mercy of unqualified and unscrupulous women, whose chief allurement to her victims is the uniform of the trained nurse.

IERNE.

